# A Critical Review of the Use of Virtual Reality in Construction Engineering Education and Training

**DOI:** 10.3390/ijerph15061204

**Published:** 2018-06-08

**Authors:** Peng Wang, Peng Wu, Jun Wang, Hung-Lin Chi, Xiangyu Wang

**Affiliations:** 1 School of Engineering and Technology, Southwest University, Chongqing 400715, China; peng.wang@curtin.edu.au; 2 Australasian Joint Research Centre for Building Information Modelling, Curtin University, Perth 6102, Australia; jun.wang1@curtin.edu.au (J.W.); xiangyu.wang@curtin.edu.au (X.W.); 3 School of Design and the Built Environment, Curtin University, Perth 6102, Australia; 4 Department of Building and Real Estate, The Hong Kong Polytechnic University, Hung Hom, Hong Kong, China; hung-lin.chi@polyu.edu.hk; 5 Department of Housing and Interior Design, Kyung Hee University, Seoul 130-701, Korea

**Keywords:** virtual reality, construction engineering, training and education, building information modelling, construction safety

## Abstract

Virtual Reality (VR) has been rapidly recognized and implemented in construction engineering education and training (CEET) in recent years due to its benefits of providing an engaging and immersive environment. The objective of this review is to critically collect and analyze the VR applications in CEET, aiming at all VR-related journal papers published from 1997 to 2017. The review follows a three-stage analysis on VR technologies, applications and future directions through a systematic analysis. It is found that the VR technologies adopted for CEET evolve over time, from desktop-based VR, immersive VR, 3D game-based VR, to Building Information Modelling (BIM)-enabled VR. A sibling technology, Augmented Reality (AR), for CEET adoptions has also emerged in recent years. These technologies have been applied in architecture and design visualization, construction health and safety training, equipment and operational task training, as well as structural analysis. Future research directions, including the integration of VR with emerging education paradigms and visualization technologies, have also been provided. The findings are useful for both researchers and educators to usefully integrate VR in their education and training programs to improve the training performance.

## 1. Introduction

Virtual Reality (VR) technologies have been rapidly recognized in construction engineering education and training (CEET) programs because they are believed to be effective in enhancing the quality of such programs. A representative taxonomy of the visualization system for positioning VR was originally made by Milgram and Colquhoum [1], and describes how “virtual” and “real” are merged in different proportions for creating a visualization environment. There are four different levels on the reality-virtuality (RV) continuum to be defined: Pure Real Presence, Augmented Virtuality (AV), Augmented Reality (AR) and Pure Virtual Presence. Strictly speaking, VR technologies are those visualization techniques referred to pure virtual presence, and nowadays are attracting much attention for improving communications in professional work and shared spaces. Benford et al. [2] introduced a classification of shared spaces based on their transportation, artificiality and spatiality. They can be categorized as media spaces, spatial video-conferencing, collaborative virtual environments, telepresence systems and collaborative augmented environment. Most of them have adopted different levels of VR involvement in recent years. There are many studies which have demonstrated the positive impact of VR in such adoptions [3,4]. Goedert et al. [4] developed a virtual interactive construction education platform which provided game-based safety training through the use of simulation and modelling. The advantages of using VR in education and training are related to its ability to enable students to interact with each other within virtual three-dimensional (3D) environments. Intuitive sense about the learning subjects can also be developed by interacting with the objects, related messages and signals in the virtual environment. Different from the conventional education and training approaches, such as the utilizations of static pictures or two dimensional (2D) drawings, VR’s visual representation allows more degrees of freedom (DoFs) to be integrated.

Since the early 2000s, various visualization techniques, such as VR and its sibling development, AR, have been adopted to enhance learning experiences. VR, as an effective tool, has proven to be effective for providing better understanding and visualization capabilities. For example, in architectural education and training, students can perceive different architectural spaces through a 3D object, rather than viewing traditional drawings. In addition, the education and training using traditional 3D approaches relies on the use of a mouse or keyboard to interact with the computer-generated structural form. However, in the VR environment, the immediate results of interactive activities, such as pulling and grabbing, can be visualized in a real-time manner [5].

Due to the rapid changes in the technologies adopted in industry, providing sufficient training programs to improve the daily activities of employees has played an important role. Traditional training programs, such as computer-based learning, are unable to equip decision makers to deal with various situations. In addition, for projects which significantly value productivity (such as oil and gas plant maintenance), on-the-job training is not possible because on-site work conditions are usually not revealed until the maintenance project begins. VR has therefore been promoted to address these practical problems in education and training.

VR has also been integrated with other enabling technologies to further enhance the performance of construction education and training. In the construction industry, there has been a rapid development of Building Information Modelling (BIM) [6,7,8,9]. One of the benefits of BIM is related to its effectiveness in improving the performance of education and training. For example, Russell et al. [10] argued that the BIM technology is useful to train students on the skillful use of 3D modelling techniques, which are believed to replace the traditional computer-aided design (CAD). Following the development of BIM, Augmented Reality (AR) is now very commonly adopted as well to support interactive visualization [11]. One major benefit of AR is the provision of engaging, motivating and immersive contents. As Chen et al. [12] pointed out, such contents are able to help students better understand their interactions with the 3D objects.

Despite the rapid development of VR and other enabling technologies, there have been limited studies on a systematic investigation of the development and its implementation in construction engineering education and training [13]. Although VR has already been adopted in architecture engineering and construction education [14,15,16,17], the use of a head-mounted display (HMD) can cause problems such as discomfort and poor depth perception [18]. To avoid these problems, portable technologies that are less immersive and present have recently been developed [19,20]. More importantly, the use of VR does not necessarily involve education and training pedagogy. It appears that there is a large research gap related to a systematic investigation on the development and use of VR in education and training [21].

The aim of this review is to conduct a comprehensive review on VR-related studies in CEET, including: (1) identifying VR and VR-related technologies and their applications; (2) investigate the implementation areas of these technologies; and (3) identify future research directions and potential benefits to help further adoption of this approach for CEET. This review is not an exhaustive analysis of all VR-related studies. However, given that it includes all peer reviewed journal articles related to VR from 1997 to 2017, it offers a useful summary of the status quo of VR in CEET. The review is organized into the following sections. Section 2 discusses the research method, including paper retrieval and the systematic analysis approaches. Section 3 summarizes the VR technologies that have been developed and used in CEET. In addition, it summarizes the implementations areas of VR technologies. Section 4 investigates the future directions of VR development and implementation in CEET. Section 5 concludes this review. 

## 2. Research Method

This study adopted a three-stage research design. A paper retrieval process related to VR research and applications in CEET was conducted. All retrieved papers were then analyzed based on the type of technologies implemented and the application areas. Results were summarized and future directions of VR research and applications in CEET were proposed.

### 2.1. Paper Retrieval

Three search criteria were established for the paper retrieval process. As the systematic review is related to investigate VR-related research and applications in CEET, only academic journal articles were selected for review, considering their relatively high impact. Conference papers, book chapters and articles in non-leading or non-international journals were not considered. Scopus and Web of Science, which were the largest two academic databases, were used for the searching. In addition, the keywords used in the retrieval process included virtual reality, virtual environment, 3D, game, construction, architecture, structural engineering education. The search rule was: (virtual reality OR virtual environment OR augmented reality OR 3D OR game) AND (education OR training). All publications which contained the above keywords in the Title/Abstract/Keywords were identified. A total of 347 articles were retrieved from 1997 to September 2017. A manual screening process was then adopted to ensure all retrieved articles were related to the aim of this study. A total of 66 publications were identified for further analysis. 

### 2.2. Data Analysis

The 66 selected publications are analyzed based on a few codes. The codes are adapted from a few similar studies using content analysis, such as Mok et al. [22]. Table 1 shows the codes used in this study.

## 3. Results

### 3.1. Overview of Selected Publications

**Figure 1:**
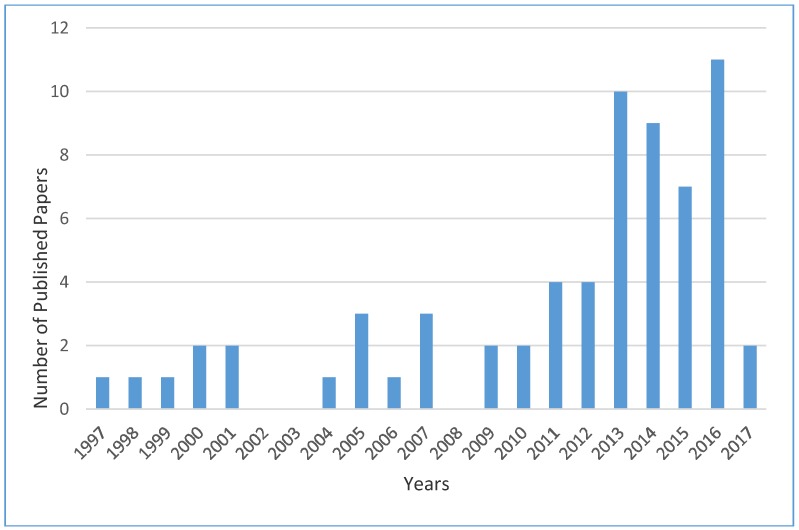
Number of publications on VR and its implementation in CEET from 1997 to September 2017.

Figure 1 shows the number of publications characterized by publication year, indicating that research interest on VR and its implementation in CEET has been increasing since 2013. Some notable publications in year 2013 are: location tracking and data visualization technology to advance construction ironworkers’ education and training in safety and productivity [23], which presented a novel real-time location tracking and data visualization in worker training environment, and *A framework for construction safety management and visualization system* [24], which proposed a framework for visualization system to enhance capacity of workers on construction site.

Table 2 presents the distribution of selected publications characterized by the publication venues. Over 24 journals containing articles related to VR in CEET were identified. As can be seen from Table 2, Journal of Professional Issues in Engineering Education and Practice and Automation in Construction are the most two popular venues for VR in CEET. Some notable publications in the Journal of Professional Issues in Engineering Education and Practice are: *Use of tangible and augmented reality models in engineering graphics courses* [12], and *BIM-enabled virtual and collaborative construction engineering and management* [25].

### 3.2. Technologies

VR and related technologies in CEET can be categorized into five major types, including desktop-based VR, immersive VR, 3D game-based VR, BIM-enabled VR and Augmented Reality (AR). This is categorized based on the different uses of visualization media as well as those of display platforms. The focus of the study is placed on the observations of related VR technology developments and their evaluations under CEET programs. It should be noticed that the categorization is enumerated, but does not limit further considerations covering all perspectives related to VR, including hardware, software, visualization and interaction issues. The detailed taxonomies of VR, as well as virtual environment systems, can be referred to in Milgram and Colquhoum [1], and Hale and Stanney [26]. Table 2 presents the distribution of the selected publications characterized by the technologies that are adopted. As can be seen from Table 3, the most commonly adopted VR systems in the literature are BIM-based VR and desktop-based VR, accounting for 47% and 26%, respectively. However, while the development of desktop-based VR is relatively stable, the development of BIM-based VR technology and AR has attracted much attention in recent years, with 27 and 7 publications respectively. 

#### 3.2.1. Desktop-Based VR

Desktop-based VR is the most commonly adopted VR technology in CEET in the early stages. As can be seen from Table 3, 6 of the 7 studies from 1997–2001 are related to desktop-based VR. According to Chen et al. [27], the technology uses a simple computer monitor as the platform for accommodating virtual activities. Desktop-based VR displays a 3D virtual world on a desktop screen without any tracking equipment to support. It relies on the users’ spatial and perception abilities to experience what happens around them. Most of the tasks can be conducted through the use of mouse and keyboards. As the technology only relies on the use of monitors, keyboards and mouse, it is considered to be relatively cheap when compared with other technologies.

Some of the most notable developments of desktop-based VR are the V-REALISM [28] and the Interactive Construction Management Learning System (ICMLS), developed by Sawhney et al. [29]. V-REALISM is developed for maintenance engineering training. It uses Computer-Aided Design (CAD) to construct the geometrical models which are then displayed through the OpenGL programming interface. V-REALISM adopts a hierarchical structure for the geometrical models which can facilitate the navigation and operation of the models in the virtual environment. This is considered to be one of the major contributions. Similarly, ICMLS was developed to address the disconnection between education and real-life on-site operations related to the use of construction equipment and methods. According to Sawhney et al. [29], ICMLS is a web-based system which relies on the creation of virtual models through virtual reality modelling language (VRML) and the demonstration of appropriate operations through discrete-event simulations (DES) and web-based computing. According to Mawlana et al. [30], ICMLS can clearly provide the needs of on-site construction which can then be embedded into CEET. The development of desktop-based VR is relatively stable, with recent developments focusing on 3D computer models and virtual laboratory to improve students’ motivation and comprehension [31,32].

#### 3.2.2. Immersive VR

Compared with desktop-based VR, immersive VR relies on the use of special hardware, such as the head-mounted device (HMD) and sensor gloves, to withdraw users from the physical world and provide an immersive environment. Spatial immersion is created by surrounding with images, sounds or other virtual scenarios, user can feel the virtual world is “authentic” and “real.” A typical demonstration of immersive VR is provided in Waly and Thabet [33], who developed the Cave Automatic Virtual Environment (CAVE). An immersive virtual environment is created around the position of the user’s location. As the position of the user changes, his/her position in the virtual environment also changes. In addition, various sensors can be embedded in the accessories of the participants, e.g., the gloves and suits to offer real-time feedback [34,35,36]. Due to the real-time capabilities, immersive VR is believed to be advantageous over the desktop-based VR system [37]. 

Another typical immersive VR system is the virtual structural analysis programme (VSAP), developed by the Virginia Polytechnic Institute and State University [38]. According to Setareh et al. [38], the main use of the system is to understand the structural behavior of buildings in a virtual environment. The main contribution of VSAP is the development of a portal immersive interface because the traditional immersive interfaces have high cost and while desktop interface has low cost, it sacrifices the quality. An adapted Virginia Tech CAVE (VT CAVE) was therefore developed with a 3 m × 3 m × 2.75 m cubic room. VT CAVE is proved to be effective in terms of usability. 

In order to provide immersive feelings to the users, immersive VR can have more supportive control tools especially tracking equipment for interactions, such as game controllers and motion tracking devices. They are commonly adopted to detect and demonstrate the movements of subjects in the virtual environment. Sacks et al. [37] used a 3D immersive VR power-wall for construction safe training education. The setting of the power-wall consisted of three rear-projection screens, and it is an open configuration of three-sided CAVE that uses 3D stereo projection with active glass. The trainees used a head tracking system and XBOX controller [39] that was also tracked using eight cameras mounted on the tops of the screens. Three software tools were used, the building demonstrated in the system was modelled in Autodesk Revit [40], other 3D geometry was modelled using 3D Studio MAX [41], and the VR scenarios were generated with EON Studio v6 [42]. The results show that VR-based training was more effective in improving concentration and giving trainees a measure of control over the environment.

#### 3.2.3. 3D Game-Based VR

3D game technology, which aims to enhance user interactions, refers to computer-based game-like training scenes through integrating visual, interactive, network and multi-user operating technologies and so forth. As game-based training, it can be used to enhance collaboration and interaction among students through the provision of tasks that are useful and close to real-life operations [43,44,45,46]. Other than focusing merely on the immersive effect, game-based VR focuses more on game objects’ interactions. For example, collision reactions can be precisely described through a physics simulation module in a game engine. In 3D game-based VR, simplified collision boundary and ray tracing methods are adopted to reduce the complexity of detection processes. In this case, game objects should be defined by both their geometric properties and collision boundaries. For complex objects such as construction excavator or cranes, it helps reduce the complexity and can make “collision detection” computationally easier.

For example, Guo et al. [13] developed a game-based safety training system, which is an online platform that allows trainees to use input devices, such as keyboard, mouse and game controllers (i.e., Wii [47] in this case) and so on, to operate virtual tasks, such as equipment operation and material delivery. The main advantage of the system is related to the availability of repeated trials at a rather low cost. For example, different methods and schedules to operate the equipment can be tested through the use of the game-based approach. Through the testing, the potential issues, e.g., health and safety considerations, can be identified. In addition, Le et al. [48] developed a game-based training platform for managing construction defect. The virtual components are created through the use of Revit Architecture and close-to-reality defect scenarios are represented with the assistance of Linden Scripting Language. In this platform, the students are trained with defect knowledge. They will then be invited to identify defects and possible activities that can lead to defect in various scenarios, the test outcomes show positive in terms of interactivity and performance. 

#### 3.2.4. BIM-Enabled VR

BIM is related to the creation and use of a three-dimensional objects, which also contain relevant properties information [49,50]. The relevant properties information particularly referred to that of necessary data required in a practical building project through its entire life cycle, including design, planning, construction, operation and maintenance stages. As such, BIM-enabled VR relies on the model, emphasizing on the data binding and connections behind other than other VR categories, to simulate construction processes and operations. Visualization is one of the most important characteristics of BIM [11]. Users can access BIM data in immersive visualization environment and analyze factors like cost and material type to develop effective building design in real time. By reviewing the design details, all elements of the BIM model from architecture and structure to Mechanical, Electrical and Plumbing (MEP) can be discussed in a more detailed way. For example, BIM-enabled VR allows user to take building design into a 3D virtual environment with all relevant building information, experiencing the BIM model in a virtual environment without the restrictions of peering into a 2D drawings, and actually inspecting the design space. Tools like Autodesk Revit Live [51] allow trainees to easily move from conventional 2D drawing design scenarios to those in BIM-based VR interactive environments, maintaining the integrity of building management data in the virtual environment before the building is actually built to understand how all of the design elements will come together. One of the biggest advantages of BIM-based VR is the ability of the model to reflect real-time changes. Xie et al. [52] pointed out that traditional VR models that are created by VRML may have difficulties in incorporating real-time information. Such difficulties may be caused by the compatibility issue. In addition, many decision-making tools have also been developed to assist the decision making process. For example, Woodward et al. [53] developed a software system to combine 3D models with schedule information so as to visualize the construction work on site. Park et al. [5] developed an interactive building anatomy modelling (IBAM) system. The system enables students to interact in a VR environment with building elements. An embedded question-and-answer game can also be integrated to enhance the learning experience. 

#### 3.2.5. Augmented Reality

AR uses sensory technology to provide a live direct or indirect view of a physical environment with augmented virtual information. The sensory technology can provide sound, video or graphics. It should be noted that AR and VR are different visualization technologies. According to the evaluation by Fonseca et al. [15], compared to a VR environment, AR enables users to interact with objects (including modifying the scale, position and other properties) that fit perfectly into the real environment. As such, many studies argued that AR technology could provide new interaction possibilities and promote active student participation [54,55,56]. For example, Chen et al. [12] used ARToolKit [57] to develop the AR model to educate the students on their ability to recognize spatial objects. As the AR model is able to project different 3D models in the real environment, it can enhance students’ learning [58]. In addition, as mobile devices are becoming more convenient for learning, many applications have been developed to embed AR in mobile devices. For example, Williams et al. [59] used a mobile AR (MAR) environment to train users on context-awareness. In addition, a mobile context-aware AR tool, CAM-ART, was developed by Shirazi et al. [19] for construction engineering undergraduate course. In the CAM-ART AR platform, static extensible mark-up language is used for content definition and JavaScript logic is used to define the interactions between objects. In addition, Kim et al. [60] developed an AR-based platform to optimize construction process through adjusting equipment operation. The advantages of AR in this research are that the technology enhances visualization from operators’ perspective and surrounding constraints can be identified.

### 3.3. Categories of VR Application in Construction Engineering Education and Training

From the review, VR applications can be categorized into four groups, including: (1) architecture visualization and design education; (2) structural analysis education; (3) construction safety training and (4) equipment and operational task training (see Table 4).

#### 3.3.1. Architecture Visualization and Design Education

From the review, almost 50% of the publications about VR applications in CEET are related to architectural visualization. VR significantly helps students to understand principles of the architectural design as well as professors to explore the students’ projects to detect hidden flaws. 

Portman et al. [106] pointed out that the main benefit of using VR in architectural design is the improved graphics, details of modelling, and character modelling delivered through modelling technologies. For example, Yan et al. [107] demonstrated the use of the BIM game in the architectural design process. In the BIM game, users are able to create avatars with first- and third-person views of the real environment, and use these data to create navigation options. Another benefit of VR in architecture visualization is that it enables the comparison of different designs at the same time. For example, in the 3D interactive virtual environmental provided by Kamath et al. [108], students can explore and interact with virtual building. They can also take the CAD data of a building and convert it into a simulation, and modify the objectives as they wish in the simulation. The usage of virtual worlds in the field of architectural education can benefit students in terms of understanding essence of architecture, which can be the first step of their careers.

#### 3.3.2. Construction Safety Training

Construction safety training is the second largest application areas of VR in CEET, with 12 publications (18%). The construction industry is a high-risk industry where the accident rate remains high. Some of the reasons leading to the high risk include limited safety knowledge of on-site employees and lack of safety awareness and training of these employees. Traditionally, construction safety training is provided in a classroom setting with slide presentations or videos. The safety information provided in the presentations and videos often do not represent real construction site conditions [109,110]. There are limited interactive methods to effectively engage trainees to improve their training performance [23]. 

A few VR and related technologies, such as BIM, game technologies, and AR, have therefore been developed to improve the current construction safe training practices. For example, Pedro et al. [17] developed a virtual platform for university students to access safety information through smart devices by scanning QR codes. Although the development of the VR components and the classification of the safety information is considered to be time consuming, the results are found to be promising. Students’ motivation and engagement to learn is improved in the VR-based training. Some strategies have also been proposed to address the limitations of time. For example, the BIM objects from previous construction projects can be collected, adapted and stored into a virtual database. In order to raise real-time safety awareness, a framework for safety training and visualization system (SMVS) that integrates BIM, location tracking, AR and game technologies, is proposed by Park and Kim [24]. The system can provide workers with safety knowledge through mobile device and improve works’ safety awareness effectively. By utilizing the system, it could enhance workers’ real-time communication ability in unsafe environment. Clevenger et al. [87] developed a BIM-enabled virtual construction safety training module to evaluate the roles of 3D visualization in safety training and education in construction. It shows that BIM-enabled safety training is very effective for undergraduate students.

#### 3.3.3. Equipment and Operational Task Training

VR has also been implemented in simulating equipment and operational activities. Similarly, traditional construction training on operational activities is based on a classroom environment, followed by on-site training. Although on-site training is considered to be an important step for trainees to gain useful experience, this involves a high risk of injury to the operator and damage to the equipment. Instead, training in a VR-based environment will bring significant benefits in terms of cost and safety. As the training is based on simulation, it does not include commonly seen costs such as fuel consumption and equipment rental. In addition, as the hazardous objects can also be reflected in the virtual environment, the VR-based training can significantly reduce the risks of being exposed to any risk of harm [92]. Some notable developments related to the use of VR in equipment operations includes the multiuser virtual safety training system (MVSTS) [95], which trains employees on the dismantling procedure of tower cranes. The after-training survey indicates that such method performs better than the traditional training methods. In order to access real-time information for construction safety and operation, Cheng and Teizer [93] developed a framework that contains real-time data collection and visualization in construction, it demonstrates that vital safety and operation information can be monitored and visualized for increasing workers’ situational awareness.

#### 3.3.4. Structural Analysis

Although structural analysis is a fundamental subject in engineering, students are usually not too enthusiastic about the subject because of the high level of abstractions and the difficult of understanding the abstractions and concepts in traditional 2D drawings [105]. Young et al. [16] investigated the use of 3D visualization of structures and found that the animation process, e.g., on the stress and strain of structures, can effectively help promote students’ learning on structural analysis.

Similarly, Fiorentino et al. [101] used the AR approach to help student understand Finite Element Analysis (FEA) in structural analysis. In this approach, the FEA results are dynamically demonstrated in the real model as the students changes the properties and characteristics of the simulation. Although the use of VR has its limitations in structural analysis (e.g., the simulation time is largely affected by the complexity of the model), the technology has brought about new perspectives on the education and training of structural analysis.

## 4. Future Research Directions 

After a comprehensive review of all VR-related articles in CEET, five future research directions are proposed. The validation focuses of those future directions could be put on determining the necessity of VR-related technologies, identifying and evaluating human visualization and interaction issues, validating the abilities to the systematic integrations in future CEET scenarios.

### 4.1. Integrations with Emerging Education Paradigms

Given the observations from the previous research effort in VR-related CEET applications, none of the research been focused on the other way around; that is, on identifying suitable teaching or learning paradigms for VR environments to cope with under particular construction scenarios, neither for potential interaction issues. As a proposal for a future research direction, different VR technologies can be further evaluated through how they can be systematically integrated with emerging teaching and learning approaches, such as a recently formed education paradigm: flipped classroom [111]. A flipped classroom is one kind of learning method that requires self-learning actions from students through online teaching material during off-class time, and they thus participate in discussion and team work activities during class. The enhanced interaction between students and objects can help address the passive learning in a traditional classroom setting [75]. What VR can be expected to bring to the flipped classroom includes immersive simulation, multi-user interaction and real-time active learning. With these features, VR-enabled teaching materials can support the development of the flipped classroom to create an active and dynamic learning environment for students. Immersion and interaction are the key factors of VR and can help teachers develop interactive teaching materials for students to perform self-learning activities with sufficient engagement, and cooperative project assignments. The evaluations of integrating such emerging education paradigm with VR technologies and how such integration can benefit all stakeholders in CEET will be a worthy topic that requires further investigation.

### 4.2. Improvement of VR-Related Educational Kits

There is a significant trend in developing new VR devices in order to further enhance the level of immersion and interaction in the virtual environment and reduce the cost, size and perception burdens of human. As can be seen from the current development of VR-related CEET applications, especially equipment and operational task training, there are several mature products in the market which have been widely utilized in the research area of VR education. However, such products still face some limitations. The cost of such products may be high. For example, although CAVE can provide high-resolution images with advanced visualization as part of the high-quality display system, the cost of such a system is very high. Although the CAVE2 cost has been reduced by 50% compared with CAVE1 in recent years, it still reaches $926K [112]. 

In addition, a fully immersive system should provide a large field of view to offer users real life immersion [113]. However, a few VR technologies, such as shutter glasses, have failed to provide such a large field of view. As such, over the past few years, many studies have been conducted on using head tracking mechanisms to translate movements of the user’s head into virtual camera movements. For example, Hilfert et al. [114] showed the possibilities of naturally interacting within a virtual space using an Oculus Rift [115] head-mounted display and Leap Motion [116] hand-tracking device. Besides Oculus Rift, there are many VR glasses, such as Microsoft HoloLens [117] and HTC VIVE [118], on the market with relatively low prices and great accessibility. In addition, gesture control, such as those brought by Leap Motion, is the most intuitive way to interact with a virtual environment. In Hilfert’s [114] research, it is able to track the student’s hands in a real environment, and their movements can be mapped simultaneously in the virtual environment.

These new products have attracted great attention due to their promising capabilities of raising interaction in virtual environments. In the future, research and engineering effort in creating more effective VR toolkits will continue. As such, it seems necessary that these new technologies should be reviewed in a timely fashion for their specific applicability in CEET. For example, the increasing of immersive feeling and dynamic in the virtual environment can also cause more human dizziness when people are exposed to a virtual environment [119]. How to design engineering curriculums considering human bodies’ reactions should be investigated as a future research direction. 

### 4.3. VR-Enhanced Online Education

Based on the review of the previous research, online education is rarely discussed in CEET scenarios. In addition, it is potentially necessary, given that it fits to the nature of cooperation in a construction project, which involves multi-disciplinary roles and a considerable number of stakeholders. They need to put their effort into massive consultation, coordination and communications, which sometimes would be easier and more efficient to perform at distance or in an asynchronous way. Cooperative systems, like BIM, in particular, have nowadays become suitable visualization and interaction platforms, while the online education of construction engineering is still lacking. In recent years, online education and open universities have become increasingly popular. According to Wu et al. [120,121], online or distance learning refers to a learning environment where the students and the classrooms and the teachings are physically separated. Online learning has recorded continuously high growth rates when compared to traditional classroom learning, because it has distinctive advantages in terms of flexibility and accessibility. However, the laboratory components are still found to be difficult to be translated into an online environment, and it is still a big challenge for teachers to help students concentrate on learning through the Internet, which usually involves other distractions, such as social media and online gaming.

VR-enhanced learning has the potential to help online learners engage with the learning process given that it has been successfully employed in conventional engineering education to improve students’ spatial skill and concentration [100]. 3D virtual objects and the interactions with them can attract and maintain the users’ attention [122]. However, the implementation of immersive education in distance learning has not been fully investigated in terms of pedagogy and a systematic design of learning curriculum, especially in CEET scenarios. These are interesting topics which can be investigated in future studies.

### 4.4. Hybrid Visualization Approaches for Ubiquitous Learning Activities

Based on the reviewed publications, VR technologies demonstrate featured benefits depending on how realistic the virtual information provided in different CEET scenarios, such as heavy equipment training, design model review and site inspection. In the most of such cases, mobility and solid interactions at training field are still vital. There is a potential research direction in coming up with hybrid visualization solutions to acquire the sensation of actual presence, e.g., touching, hearing and so forth, along with virtual ones at the same time. Users are encourage to use VR technologies closely with other visualization approaches, such as AR, to create a multivariate mixed reality (MR) education environment [123]. With the rapid evolution of other educational kits for facilitating learning activities, the adoptions of mobile and context-aware devices have brought promising results in realizing ubiquitous learning environment of engineering education. With the support of wireless networks [124] and real-time sensing technologies [125], ubiquitous environments are transitioning the learning style towards one that can take place anytime and anywhere without the limitations of time and locations [126]. Ubiquitous learning environments are expected to exist everywhere not only at home, classrooms or training facilities, but also in the streets and in every corner of cities. For example, field and hands-on learning activities with real-time instructions for structure analysis of building and civil infrastructures have become possible [99]. Microsoft has started a promotion of mixed reality environment that makes users feel present in such environment where they can move, interact, and explore in the real world and receive responses in the virtual one [127]. The suitability of the integration of VR and other technologies for CEET activities should be evaluated to maximize the learning performance of students and trainees.

### 4.5. Rapid As-Built Scene Generation for Virtual Training 

Emerging scanning technologies, including reconstruction processes of laser scanned point clouds or photogrammetry [128] for captured images, support a rapid as-built modelling in the virtual world, leading cost-efficient and accurate approaches to generate actual scenes for the use of engineering training and education. The level of reality in terms of modelling accuracy, level of detail (LoD) and shading for the as-built 3D model are increasing, as are the related automation processes [129]. However, no such technologies, according to the reviewed publications, have been used for educational purposes in CEET. Other than facilitating the digitalization of buildings or facilities for construction and management purposes [130], scanning technologies can be used as learning or training materials for students or trainees to get high level of awareness about the content of learning subjects. With the support of these technologies, educators can easily develop the required virtual scenes for CEET activities. For example, it will be much easier to create a realistic and cost effective virtual scene for safety training. Learning resources can also be retrieved from the digitalization processes for BIM and Smart Cities [131].

## 5. Conclusions

In this research, a comprehensive review regarding Virtual Reality (VR) in construction engineering training and education has been conducted and the technologies, application areas and future research directions have been identified. Based on the review of 66 journal papers, the VR technologies that have been implemented in CEET include desktop-based VR, immersive VR, 3D game-based VR, BIM-enabled VR and Augmented Reality. The development of VR technologies is transitioning from desktop-based styles to mobile ones with enhanced immersion and interaction abilities. Such developments have brought benefits to many CEET topics, such as architecture design, construction health and safety, equipment operation and structural analysis. 

The contributions of this review study of the body of knowledge are threefold. It identifies domain-specific development trends of VR related applications in Construction Engineering Education and Training (CEET) practice. Based on the comprehensive literature review, immersive VR, 3D game-based VR and AR have tremendous potential to increase students’ participation, interaction and motivation. BIM-enabled VR helps students to effectively identify building in details, and it can enhance students’ spatial understanding in expandable visualized environments. In addition, future research directions have been proposed based on the observations of previous research outcomes in CEET. This review also points out the emerging trend of our development of integrated teaching support, by using VR and related visualization technologies with emerging construction information management approaches, such as Building Information Modelling (BIM). The design of VR-based educational methods should be expected to shift learning styles from teacher-centered to student-centered learning in a virtual or virtual-reality blended environment.

The review has some limitations. It covers only the technologies that are related to the CEET field. As such, it does not cover the full spectrum of the development of these technologies. The review also points out a few future research directions. The technology has not yet been fully tested on its suitability and capability with emerging engineering education paradigms, such as flipped classroom. In addition, its suitability with other emerging VR-related educational toolkits and other visualization approaches should be investigated. The development of BIM and Smart Cities can be referred to as a source which can provide useful objects to ease the creation process of virtual objects for CEET activities. It is expected that the findings of this research can be a useful reference contributing to future research or practice on implementing VR for education and training in construction and engineering.

## Figures and Tables

**Table 1:** Codes that are adopted for content analysis.

Codes	Descriptions of the codes
Publication year	The year of publication, from1997 to September 2017
Author	List of authors in the selected publication
Publication venue	The journals which accommodate the selected publication
Country	The country where the selected publication is originated
Technology	The type of VR technologies that are adopted in the selected publication
Application	Categories of VR application in the selected publication
Future direction	Future studies stated in the article

**Table 2:** Distribution of the selected journal papers by publication venues.

Journal Title	Number of Selected Papers
Journal of Professional Issues in Engineering Education and Practice	11
Automation in Construction	8
International Journal of Engineering Education	6
International Journal of Construction Education and Research	5
Computer Applications in Engineering Education	5
Electronic Journal of Information Technology in Construction	4
Journal of Information Technology in Construction	4
Practice Periodical on Structural Design and Construction	4
Journal of Architectural Engineering	2
Journal of Construction Engineering and Management	2
Engineering Design Graphics Journal	2
Journal on Educational Resources in Computing	1
Advances in Engineering Software	1
Architectural Engineering and Design Management	1
Australasian Journal of Construction Economics and Building	1
Australasian Journal of Engineering Education	1
Behaviour and Information Technology	1
Computers and Education	1
Computers in Education Journal	1
Journal of Computing in Civil Engineering	1
Journal of Engineering, Design and Technology	1
Journal of Industrial Technology	1
Materials and Structures	1
Simulation	1
Total	66

**Table 3:** The distribution of publications characterized by technology and publication year.

Research Theme	Period				Total	Percentage
	1997–2001	2002–2006	2007–2011	2012–2017		
Desktop-based VR	6	3	3	5	17	26%
Immersive VR	1	1	1	1	4	6%
3D game-based VR	0	0	0	4	4	6%
BIM-based VR	0	0	4	27	31	47%
Augmented Reality	0	0	3	7	10	15%
Total	7	4	11	44	66	100%

**Table 4:** The distribution of publications characterized by VR application in CEET.

VR Applications	Representative Studies	Frequencies
Architecture Visualization and Design Education	[5,10,12,19,25,32,37,46,54,61,62,63,64,65,66,67,68,69,70,71,72,73,74,75,76,77,78,79,80,81,82,83]	32
Construction Safety Training	[13,17,23,24,44,48,84,85,86,87,88,89]	12
Equipment and Operational Task Training	[4,60,88,90,91,92,93,94,95,96,97,98]	12
Structural Analysis Education	[16,31,38,99,100,101,102,103,104,105]	10
Total		66
